# Perception of availability and ease of access to COVID-19 vaccination in Nigeria: a cross-sectional study

**DOI:** 10.11604/pamj.2025.52.106.46930

**Published:** 2025-11-13

**Authors:** Sidney Sampson, Sunday Atobatele, Oluwafisayo Ayodeji, Adebisi Adenipekun, Saheed Dipo Isiaka, Stephen Olabode Asaolu, Gab-Deedam Shiva, Emmanuella Nzeribe, Olugbemisola Samuel

**Affiliations:** 1 Sydani Group, Federal Capital Territory, Abuja, Nigeria,; 2 Sydani Institute for Research and Innovation, Sydani Group, Federal Capital Territory, Abuja, Nigeria

**Keywords:** Perception, COVID-19, vaccination, COVID-19 vaccine, cross-sectional study, Nigeria

## Abstract

**Introduction:**

the government of Nigeria, through concerned agencies/authorities, is ensuring a large-scale and equitable distribution of COVID-19 vaccination across the country. To understand how the eligible population accesses these vaccines, we assessed the perception of availability and accessibility of the COVID-19 vaccines in Nigeria.

**Methods:**

the study was part of a larger cross-sectional survey conducted in Nigeria between July and August 2021 to understand broader behavioral, social, and access-related drivers of COVID-19 vaccines among healthcare workers (HCW) and non-healthcare workers (NHCWs) using a data tool adapted from the World Health Organization (WHO) guidance on behavioral and social drivers of vaccination. Data was collected from 1548 respondents across 8 Nigerian states using a multistage sampling approach and analyzed descriptively and inferentially, using SPSS Version 20 to understand the perceptions of vaccine availability and accessibility.

**Results:**

individual perceptions on the availability of vaccines were significant across two categories (that is, for NHCWs and HCWs) across rural and urban areas (X^2^=14.121, p<0.001) and between NHCWs and HCWs (X^2^=23.508, p<0.001). Non-health care workers were significantly more likely to perceive difficulties in accessing COVID-19 vaccines compared to health care workers (X^2^=29.8, p<0.001), and rural residents reported more challenges than their urban counterparts (X^2^=23.0, p<0.001).

**Conclusion:**

the study found that respondents' perceptions of vaccine availability and accessibility were mostly influenced by location and recommended more vaccination points across rural and urban communities to improve the COVID-19 vaccination experience.

## Introduction

The coronavirus disease (COVID-19) vaccine was first introduced in March 2021 in Nigeria by the National Primary Health Care Development Agency (NPHCDA) [1]. The government of Nigeria, through the NPHCDA and in collaboration with the State Primary Healthcare Boards (SPHCB) ensured a large-scale, equitably accessible, and proper distribution of COVID-19 vaccines across the country in an effort to mitigate the spread of the virus and to improve the health outcomes for those who will be infected [2]. This was done by bringing the COVID-19 vaccine as close as possible to the people, through ensuring its availability in various healthcare facilities across the country (rural and urban locations) [2,3]. However, this has not resulted in the massive uptake of the vaccine as expected. As of the time of this study, the reported number of vaccinated Nigerians according to the NPHCDA showed that only 2,265,805, representing just over 1% of the target population, have been vaccinated [4], with several studies attributing the low uptake of the COVID-19 vaccine to several factors [5-20]. Although the NPHCDA deployed the Traditional, Electronic self-registration, assisted electronic registration, Concomitant e-registration, and House-to-House Registration (TEACH) strategy to simplify the process to reach its goal of delivering COVID-19 vaccines to the eligible population, this was still greeted with low uptake of the vaccine [5]. Asides from the various known factors such as myths and misconceptions surrounding COVID-19 vaccine production [6-8], socio-demographic factors [9,10], and good knowledge of COVID-19 vaccination [2,11] that have been reported to influence the uptake of COVID-19 vaccination [3,12-19], the distribution and accessibility of the COVID-19 vaccines can also influence low COVID-19 vaccine uptake [20]. According to the study by Suarez-Alvarez and Lopez-Menendez [21], low rates of COVID-19 vaccination in developing countries are due to weak health systems, which resulted in inequitable access and availability of the COVID-19 vaccine. Although the government of Nigeria, through the NPHCDA, SPHCB, and other partners, is striving to ensure the equitable distribution of COVID-19 vaccines, the low rate of COVID-19 vaccination in the country could be a result of limited access and availability. Separate in-country studies have also pointed to the availability and access to COVID-19 vaccines among others as part of the barriers to vaccine uptake, especially among rural and urban dwellers, healthcare workers (HCW), and non-healthcare workers (NHCW) [2,22]. To date, no country-wide study has been conducted to understand the availability and accessibility of the COVID-19 vaccines in Nigeria based on location (rural and urban) and based on profession (healthcare and non-healthcare) workers. This study, therefore, seeks to assess the perception of vaccinees on the availability and accessibility of COVID-19 vaccines in Nigeria.

## Methods

**Study design:** the study was part of a cross-sectional survey on understanding behavioral, social, and access-related drivers of COVID-19 vaccination conducted in Nigeria between July and August 2021.

**Study setting:** six out of the 36 states (Bauchi in North-East, Kogi in North-Central, Jigawa in North-West, Bayelsa in South-South, Ondo in South-West, and Anambra in South-East) with the highest COVID-19 cases in each of the six geopolitical zones of Nigeria were selected for the study. Additionally, Lagos state and the Federal Capital Territory (FCT) were purposively selected based on the severity of the pandemic and their importance as commercial and federal capital cities, respectively.

**Study participants:** the study sample comprised healthcare workers and non-healthcare workers who had been vaccinated with the COVID-19 vaccine during the time of the assessment. The healthcare workers captured in the study were doctors, nurses, laboratory attendants, technicians, community health and extension workers, traditional medicine practitioners, and pharmacists, while non-healthcare workers are people working in the communications, education, transportation, logistics, paramilitary, and commercial sectors.

**Study size:** the sampling size for each state was obtained using the Cochran formula based on an alpha level value of 1.96 at a 95% Confidence Interval, resulting in a total of 3,493.

**Sampling strategy:** the respondents for the study were then selected using a multistage sampling approach. With each state further divided into local government areas (LGAs), the state´s micro plan was used to disaggregate the LGAs into rural and urban, from where one rural and one urban LGAs were randomly selected for the study. Respondents were then recruited for the study in each LGA through the snowball method. The study adopted the snowball method because of the pandemic and the difficulty involved in getting the target audience due to lockdown measures imposed to curb the spread of COVID-19. The snowball sampling procedure was executed by first identifying initial participants, who were community leaders, healthcare workers at local primary health centers, or known residents who met the eligibility criteria (adults aged 18 years and above residing in the selected LGAs who have taken the COVID-19 vaccine). A diverse group of 5-8 initial participants was purposively selected in each LGA to initiate recruitment from varied social networks. After providing informed consent and completing the study survey, each initial participant was asked to assist in the study by referring up to two or three other individuals from their community (friends, family members, or neighbors) who also met the eligibility criteria. These referred individuals were then contacted by the research team, screened for eligibility, and, upon consent, enrolled in the study. After their participation, the same referral process was repeated with them, creating expanding chains of referrals. This process continued iteratively until the predetermined sample size for each LGA was achieved. To ensure diversity, we monitored the socio-demographic characteristics (age, gender, education) of incoming participants and, if necessary, identified new participants from underrepresented groups to initiate new referral chains.

**Exclusion criteria:** communities with pockets of violence and other security issues were excluded from the study. Persons under 18 years old who were not eligible to take COVID-19 vaccines during the period of the study were also excluded. In alignment with standard study ethics, individuals who refused to consent to participate were excluded.

**Data collection:** the data collection tool was adapted from the WHO behavioral and social drivers´ tool, which is structured into four domains: thinking and feeling, motivation, social processes, and practical issues [23]. Perceived availability and perceived ease of access to the COVID-19 vaccine were captured under the practical issues domain. The possible responses to perceived availability were “ Readily available”, “Not readily available”, and “I do not know”, while the possible responses to perceived ease of access were “Not Easy”, “Moderately”, and “Very Easy”. The data collection tool was programmed on KoboCollect and deployed using a mobile phone. The data tool was piloted and revised based on feedback from pre-testers. The data tool was then administered to the respondents by research assistants who had undergone face-to-face training for data collection, adhering to all COVID-19 protocols, including social distancing and the use of face masks.

**Data analysis:** of the 3,493 total respondents, the analysis was restricted to the 1,548 vaccinated respondents who answered questions on vaccine access and availability. This analysis was guided by a conceptual framework wherein sociodemographic and occupational factors (independent variables) are hypothesized to influence an individual's perception of health services (dependent variables). The specific research questions addressed were: i) is there a significant association between a respondent's location (rural vs. urban) and their perception of the ease of access to COVID-19 vaccines? ii) is there a significant association between a respondent's profession (healthcare worker vs. non-healthcare worker) and their perception of the availability of COVID-19 vaccines?

**Independent variables:** location (rural/urban) and profession (healthcare worker/non-healthcare worker).

**Outcome measures:** perceived ease of accessibility and perceived availability of COVID-19 vaccines. The sociodemographic characteristics of this sample are presented using descriptive statistics (frequencies and percentages). To assess the association between the perception of vaccine availability and ease of access and location (rural/urban) as well as profession (HCW/NHCW), we performed chi-square tests. Data analysis was conducted using SPSS version 20.0 (IBM Corp., Armonk, NY), and a p-value less than 0.05 is considered statistically significant. The results of the data analysis were presented in tables.

**Ethical approval:** the guidelines on research involving human subjects, according to the Helsinki Declaration, were followed. The survey was anonymous, and information confidentiality was guaranteed, with verbal consent obtained from participants. The study protocol was also approved by the National Health Research Ethics Committee (NHREC) in the Federal Ministry of Health (FMoH), Abuja: NHREC/01/01/2007-14/07/2021. Approval was also received from the U.S. CDC´s Center for Global Health Human Subjects Office as a public health activity.

## Results

**Socio-demographic characteristics of respondents:** responses from a total of 1548 respondents who were vaccinated were analyzed. Female respondents were just about half (n=781; 50.5%) of the total respondents. The study sample consisted of 1063 (68.7%) HCW and 485 (31.3%) NHCW. Most of the respondents (n=1090; 70.4%) lived in urban areas rather than in rural areas (n=458; 29.6%). Slightly above one-third (n=553; 35.7%) were between 30-39 years, as over a quarter (n=406; 26.2%) of them were between the ages of 40 and 49, while nearly a quarter (n=376; 24.3%) of the respondents fell between 20-29 years. Others are as shown in Table 1. For the religion of the respondents, 3 out of 5 (n=941; 60.8%) of the study participants were of Christian faith, while nearly 2 out of 5 (n=602; 38.9%) respondents were of the Islamic faith, and two people each were of traditional faith and other forms of religion. More than half (n=881; 56.9%) of the study respondents have completed their undergraduate degrees, and over one-tenth (n=212; 13.7% and n=175; 11.3%) of the respondents have only completed secondary school level of education and a postgraduate degree, respectively. A limited proportion (n=135; 8.7%) of the study respondents had only attained primary school education, while very few (n=67; 4.3%, n=40; 2.6%, and n=37; 2.4%) of the respondents had a Technical College education, a graduate degree, or no formal education.

**Table 1:** socio-demographic characteristics of the respondents surveyed about availability and ease of access for COVID-19 vaccines in Nigeria, from July to August, 2021 (n=1548)

Socio-demographics	Category	Frequency	Percentage
Gender	Male	767	49.5
	Female	781	50.5
Age (years)	18-19	12	0.3
	20-29	376	24.3
	30-39	553	35.7
	40-49	406	26.2
	50-59	181	11.7
	60 Above	5	0.8
Group	Healthcare workers	1063	68.7
	Non-healthcare workers	485	31.3
Location	Rural	458	29.6
	Urban	1090	70.4
Religion	Christianity	941	60.8
	Islam	602	38.9
	Traditional	2	0.1
	Others	2	0.1
Education	No formal education	37	2/4
	Primary school	135	8.7
	Secondary school	2122	13.7
	Technical college	67	4.3
	Undergraduate	881	56.9
	Graduate	40	2.5
	Postgraduate	175	11.3

**Perception of the respondents on the availability of the COVID-19 vaccines:** the analysis revealed that a respondent's profession was a significant factor in their perception of vaccine availability, whereas their geographical location (rural vs. urban) was not. A strong and statistically significant association was observed between professional status and the perception of COVID-19 vaccine availability (X^2^= 23.51, p<0.001). Overall, healthcare workers (HCWs) held a more favorable view of vaccine availability compared to non-healthcare workers (NHCWs). Specifically, a majority of both groups perceived the vaccine as readily available; however, this perception was more prevalent among HCWs (60.9%, n=847) than among NHCWs (56.9%, n=276). A more notable difference emerged in the negative and uncertain response categories. The proportion of NHCWs who perceived the vaccine as not readily available (32.6%, n=158) was slightly lower than that of HCWs (35.0%, n=372). More strikingly, NHCWs were more than twice as likely as HCWs to express uncertainty about vaccine availability (10.5% vs. 4.1%, respectively). This disparity in perception between professional groups was consistent when analyzed within rural and urban subgroups, and both subgroup analyses were statistically significant (Rural: X^2^= 14.12, p < 0.001; Urban: X^2^= 26.7, p < 0.001). Interestingly, the data suggest a contextual effect of location on these professional perceptions. In rural areas, a higher proportion of NHCWs reported vaccine availability (63.9%, n=163) than HCWs did (53.7%, n=109). Conversely, in urban areas, the pattern flipped, with a substantially higher proportion of HCWs reporting availability (62.6%, n=538) compared to NHCWs (49.1%, n=113). This indicates that the experience of availability differed for each profession based on their setting. In contrast to profession, no significant association was found between geographical location and the perception of vaccine availability (X^2^= 0.459, p = 0.795). The perceptions among rural and urban dwellers were remarkably similar. Nearly identical proportions of rural (59.4%, n=272) and urban (59.7%, n=651) respondents believed the vaccine was readily available. This similarity extended to perceptions of unavailability (Rural: 33.8%, n=155; Urban: 34.4%, n=375) and uncertainty (Rural: 6.8%, n=31; Urban: 5.9%, n=64). A comprehensive summary of all response frequencies and percentages, stratified by both profession and location, is presented in Table 2.

**Table 2:** association between the perception of COVID-19 vaccine availability and respondent characteristics (profession and location) among vaccinated respondents, from July to August, 2021 (n=1,548)

		Perceived availability of COVID-19 Vaccine				χ2	P-value
		Readily available n(%)	Not readily available n(%)	I Do not know n(%)	Total n(%)		
Rural	NHCW	163 (63.9)	69 (27.1)	23 (9)	255 (100)	14.12	<0.001
	HCW	109 (53.7)	86 (42.4)	8 (3.9)	203 (100)		
Urban	NHCW	113 (49.1)	89 (38.7)	23 (12.2)	230 (100)	26.70	<0.001
	HCW	538 (62.6)	286 (33.2)	36 (4.2)	860 (100)		
Total	NHCW	276 (56.9)	158 (32.6)	51 (10.5)	485 (100)	23.51	<0.001
	HCW	847 (60.9)	372 (35)	44 (4.1)	1063 (100)		
	Rural	272 (59.4)	155 (33.8)	31 (6.8)	458 (100)	459	0.795
	Urban	651 (59.7)	375 (34.4)	64 (5.9)	1090 (100)		

X2: chi-square; n: frequency; %: percentage; NHCW: non-healthcare worker; HCW: healthcare worker

**Perception of the respondents on the ease of access to the COVID-19 vaccines:** the perception of ease of access to COVID-19 vaccines was significantly associated with both profession and geographical location (all p-values ≤ 0.001, Table 3). Overall, perceptions of difficulty were prevalent. Nearly half of all respondents found access not easy (NHCWs: 51.1%, n=248; HCWs: 47.6%, n=506). However, a clear gradient emerged based on profession. Healthcare workers (HCWs) reported a more positive perception of access than non-healthcare workers (NHCWs). While a similar proportion of both groups found access not easy, a substantially higher proportion of HCWs found access very easy (42.5%, n=452) compared to NHCWs (31.0%, n=150). Conversely, NHCWs were more likely to report only moderately easy access (17.9%, n=87) compared to HCWs (9.9%, n=105). A strong association was also found between location and perception of access (p ≤ 0.001). Urban dwellers held a significantly more negative view than rural dwellers. A majority of urban respondents (57.3%, n=516) perceived access as not easy, compared to just over half of rural respondents (52.0%, n=239). This negative perception in urban areas coincided with a much lower proportion reporting moderately easy access (5.0%, n=54 vs. 17.2%, n=78 in rural areas). The proportion of respondents finding access very easy was higher among urban dwellers (42.2%, n=460) than rural dwellers (31.0%, n=142), suggesting a polarized distribution of experiences in urban settings. The relationship between profession and location revealed nuanced patterns. The trend of HCWs perceiving better access than NHCWs held in both settings, but the magnitude of this professional disparity was greater in urban areas. In rural areas, the gap between HCWs and NHCWs reporting very easy access was large (41.4% vs. 22.4%). In urban areas, this gap was even more pronounced, with HCWs being far more likely to report very easy access than NHCWs (42.8% vs. 40.0%). Furthermore, urban HCWs were the most likely group to report very easy access (42.8%, n=388), while urban NHCWs were among the most likely to report access as not easy (42.2%, n=97).

**Table 3:** association between the perception of ease of access to COVID-19 vaccines and respondent characteristics (profession and location) among vaccinated respondents, from July to August, 2021 (n=1,548)

		Perceived ease of access of COVID-19 vaccine				χ2	p-value
Rural		**Not easy n(%)**	**Moderately n(%)**	**Very easy n(%)**	**Total n(%)**		
	NHCW	151 (59.2)	46 (18.1)	58 (22.7)	255 (100)	18.8	<0.001
	HCW	87 (42.9)	32 (15.7)	84 (41.4)	203 (100)		
Urban	NHCW	97 (42.2)	41 (17.8)	92 (40)	230 (100)	17.1	< 0.001
	HCW	419 (48.7)	73 (8.5)	388 (42.8)	860 (100)		
Total	NHCW	248 (51.1)	87 (17.9)	150 (31)	485 (100)	29.8	< 0.001
	HCW	506 (47.6)	105 (9.9)	452 (42.5)	1063 (100)		
	Rural	238 (52)	78 (17)	142 (31)	458 (100)	23.0	< 0.001
	Urban	516 (57.3)	54 (0.5)	460 (42.2)	1090 (100)		

X2: chi-square; n: frequency; %: percentage; NHCW: non-healthcare worker; HCW: healthcare worker

## Discussion

The study aimed to investigate the perception of the vaccinees on the availability and ease of access to COVID-19 vaccination. The study respondents were spread across different socio groups, and this is an indication that the snowball method of sampling adopted for the study ensured that various subgroups were fairly represented. From the result of the study, most of the respondents (both HCWs and NHCWs) in rural areas perceived the COVID-19 vaccine as readily available, and this was most likely based on their experience at various COVID-19 vaccination points they visited where they got vaccinated without having to go through much hassle. Also, this perception could probably be ascribed to the effort of the government and concerned authorities, which ensured equitable distribution of the COVID-19 vaccine. Although the perception of vaccines on the availability of vaccines was significant for NHCW and HCW in rural areas, this could probably be attributed to the fact that the agents/channels of community sensitization and spread of information in rural communities are highly influential in achieving their goals of communicating to their residents´ vital information that is considered useful on the path of the rural populace. More HCWs perceived the COVID-19 vaccine as readily available compared to NHCWs in urban areas, and this could probably be because HCWs are privy to information regarding vaccine availability, as the COVID-19 vaccination points are in health facilities. This may also be because NHCWs who might have visited vaccination points could have been asked to come back due to the unavailability of the COVID-19 vaccine at one time or the other. Generally, the study showed that the perception of the availability of the COVID-19 vaccine was higher among HCWs than NHCWs and this could probably be an indication of the privilege of vaccine information among HCWs compared to the NHCWs in both rural and urban areas. Additionally, the reduced differences that exist in percentages between vaccinated NHCWs and vaccinated HCWs can account for the small gap in the perception of the availability of the COVID-19 vaccine. In essence, this suggests that their experience at the vaccination sites informed their knowledge of COVID-19 vaccine availability. This is similar to the findings of a study that found that good knowledge about vaccine information, as regards availability, helps an individual to know when to access COVID-19 vaccination points and when not to [24]. The study of Obarisiagbon and Mokogwu [22], on the other hand, showed that poor knowledge of COVID-19 vaccine points had negatively influenced COVID-19 vaccination among traders at the Edaiken Market in Benin City. Additionally, the study found similarities in the perception of COVID-19 vaccination availability both amongst rural and urban dwellers. Although the urban dwellers were more represented in the study, the similarities in perception could probably be a result of the motivation of the respondents to get vaccinated at both the rural and urban levels. This supports the findings of El-Elimat *et al*. [25] and Shekhar *et al*. [26], where participants were willing to be vaccinated as long as the COVID-19 vaccine was available to intending recipients.

On the ease of access to the COVID-19 vaccine, the study found that a higher proportion of NHCWs in rural areas perceived ease of access to COVID-19 vaccination as not easy, contrary to the higher proportion of HCWs in rural areas who perceived ease of access to the COVID-19 vaccine as moderately easy or very easy. This might not be unconnected with the fact that COVID-19 vaccination centers are in health facilities, especially in rural areas where the Primary Healthcare Centers (PHC) solely housed COVID-19 vaccination points. As such, HCWs in rural areas can access COVID-19 vaccination at work compared to NHCWs who may have to skip a whole day of work to come into the PHCs to access COVID-19 vaccination. Moreso, in rural areas, some communities are far from PHC facilities, hence people may have to travel far distances, and through difficult terrain to access COVID-19 vaccination. This is similar to the findings of Adane *et al*. [27], who reported that 3 out of 5 HCWs who participated in their study had a good perception of COVID-19 vaccination. Contrastingly is a similarity in the perception of NHCWs and HCWs in urban areas on the ease of access to COVID-19 vaccination, where close to half of the respondents believed that there was substantial ease of access to the COVID-19 vaccination. This could be attributed to the presence of more COVID-19 vaccination centers in urban areas. At the beginning of the COVID-19 vaccination, the focus was more on urban areas due to higher COVID-19 caseloads, hence the more vaccination points. Moreso, healthcare facilities in urban areas are closer to cold chain equipment that stores COVID-19 vaccines. Also, there was more organization of COVID-19 vaccination in urban areas, with various social groups embarking on massive sensitization campaigns [28]. Additionally, the study found a slight difference in the perception of ease of access to the COVID-19 vaccine between rural and urban dwellers, as rural dwellers were more convinced of the ease of access to the COVID-19 vaccination than urban dwellers. This difference can be associated with the disparity in the population of the respondents on both ends of rural and urban areas, as well as the perception of the vaccines on the overall population of their respective areas. In furtherance, the way of life across both regions plays a pivotal role in the perception of COVID-19 accessibility. In essence, communal living is usually defined as simple, and as such, members of rural communities can easily create time to visit the vaccination site, depending on the availability of the vaccine in vaccination sites [29]. Contrarily, the urban lifestyle in many cases, particularly in Nigeria, is usually considered as complicated because of the various commitments of the urban dwellers, and this may lead to a form of an impediment for urban dwellers to visit vaccination sites to get vaccinated [30].

**Recommendation:** there is a need to explore and identify channels that can efficiently enhance the consistent communication of vital information across the board. There is also a need to ensure equitable distribution of vaccination points and communicate these vaccination points to the populace through various advocacy and social mobilization channels.

**Data availability:** the datasets used and/or analyzed during the study are available from the corresponding author upon reasonable request.

## Conclusion

COVID-19 vaccination and vaccination exercise in Nigeria is a multifaceted phenomenon, and as such, issues around the vaccine in the country can arguably be considered intricate. Findings from this study have revealed that, on the one hand, both NHCWs and HCWs in rural areas are well-informed about the availability and accessibility of the COVID-19 vaccination in their various communities. This indicates the presence of the sociological concept of collective conscience; wherein members of communities engage in communal living and share the spirit of togetherness in the community. On the other hand, there is a relative disposition to the perception of NHCWs and HCWs in urban areas on the availability and accessibility of the public to COVID-19 vaccination. Therefore, this study concluded that the respondents' perceptions of the availability and accessibility of the COVID-19 vaccine were mostly influenced by where they lived. As such, more vaccination points are needed across rural and urban communities for a better COVID-19 vaccination experience.

### What is known about this topic

- The COVID-19 vaccine has been introduced in Nigeria;
- The Nigerian national primary healthcare development agency deployed the TEACH strategy to achieve the vaccination of eligible Nigerians;
- The uptake of the vaccine has not been as massive as anticipated leading to low vaccination rates is as a result of weak health systems.

### What this study adds

- Perceptions of vaccine availability varied by geography: rural residents more often reported that vaccines were present in their communities but harder to access, while urban residents perceived better access;
- Socio-demographic characteristics such as education and occupation influenced how respondents perceived both the availability and accessibility of COVID-19 vaccines;
- Overall, accessibility challenges, rather than availability alone, emerged as the more consistent barrier shaping perceptions of COVID-19 vaccination in Nigeria.

## References

[1] Nomhwange T, Wariri O, Nkereuwem E, Olanrewaju S, Nwosu N, Adamu U (2022). COVID-19 vaccine hesitancy amongst healthcare workers: an assessment of its magnitude and determinants during the initial phase of national vaccine deployment in Nigeria. EClinicalMedicine.

[2] Egbuniwe MC, Dankano NE, Nnamani CP, Azubuike PC, Obidile VC, Ekwebene OC (2021). COVID-19 vaccine knowledge and acceptability among healthcare providers in Nigeria. immunity.

[3] Adigwe OP (2021). COVID-19 vaccine hesitancy and willingness to pay: Emergent factors from a cross-sectional study in Nigeria. Vaccine X.

[4] Reliefweb Nigeria: iMMAP/DFS COVID-19 Situation Analysis (June 2021).

[5] Chukwuocha UM, Emerole CO, Iwuoha GN, Dozie UW, Njoku PU, Akanazu CO (2022). Stakeholders' hopes and concerns about the COVID-19 vaccines in Southeastern Nigeria: a qualitative study. BMC Public Health.

[6] Chavda VP, Chen Y, Dave J, Chen ZS, Chauhan SC, Yallapu MM (2022). COVID-19 and vaccination: myths vs science. Expert Rev Vaccines.

[7] Lamptey E, Senkyire EK, Dorcas S, Benita DA, Boakye EO, Ikome T (2022). Exploring the myths surrounding the COVID-19 vaccines in Africa: the study to investigate their impacts on acceptance using online survey and social media. Clin Exp Vaccine Res.

[8] Chutiyami M, Salihu D, Bello UM, Winser SJ, Gambo AA, Sabo H (2022). Are Fear of COVID-19 and Vaccine Hesitancy Associated with COVID-19 Vaccine Uptake? A Population-Based Online Survey in Nigeria. Vaccines (Basel).

[9] Adedeji-Adenola H, Olugbake OA, Adeosun SA (2022). Factors influencing COVID-19 vaccine uptake among adults in Nigeria. PLoS One.

[10] Olomofe CO, Soyemi VK, Udomah BF, Owolabi AO, Ajumuka EE, Igbokwe CM (2021). Predictors of uptake of a potential COVID-19 vaccine among Nigerian adults. Medrxiv.

[11] Akinyemi PA, Fajobi O, Owoade IA, Elugbaju OT, Wuraola FO (2021). Community perception and determinants of willingness to uptake COVID-19 vaccines among residents of Osun State, South-West Nigeria. Int J Community Med Public Health.

[12] Alhassan RK, Owusu-Agyei S, Ansah EK, Gyapong M (2021). COVID-19 vaccine uptake among health care workers in Ghana: a case for targeted vaccine deployment campaigns in the global south. Hum Resour Health.

[13] Oguntayo R, Olaseni AO, Ogundipe AE (2021). Hesitancy prevalence and sociocognitive barriers to coronavirus vaccinations in Nigeria. Eur Rev Appl Sociol.

[14] Oriji PC, Allagoa DO, Obagah L, Tekenah ES, Ohaeri OS, Atemie G (2021). Perception about COVID-19 vaccine among patients at the federal medical centre, Yenagoa, South-South Nigeria. Int J Res Med Sci.

[15] Alya WA, Maraqa B, Nazzal Z, Odeh M, Makhalfa R, Nassif A (2022). COVID-19 vaccine uptake and its associated factors among Palestinian healthcare workers: expectations beaten by reality. Vaccine.

[16] Doran J, Seyidov N, Mehdiyev S, Gon G, Kissling E, Herdman T (2022). Factors associated with early uptake of COVID-19 vaccination among healthcare workers in Azerbaijan, 2021. Influenza Other Respir Viruses.

[17] Jennings W, Stoker G, Bunting H, Valgarðsson VO, Gaskell J, Devine D, McKay L, Mills MC (2021). Lack of trust, conspiracy beliefs, and social media use predict COVID-19 vaccine hesitancy. Vaccines (Basel).

[18] Moucheraud C, Phiri K, Whitehead HS, Songo J, Lungu E, Chikuse E (2023). Uptake of the COVID-19 vaccine among healthcare workers in Malawi. Int Health.

[19] Njoga EO, Mshelbwala PP, Abah KO, Awoyomi OJ, Wangdi K, Pewan SB (2022). COVID-19 vaccine hesitancy and determinants of acceptance among healthcare workers, academics and tertiary students in Nigeria. Vaccines (Basel).

[20] Rydland HT, Friedman J, Stringhini S, Link BG, Eikemo TA (2022). The radically unequal distribution of Covid-19 vaccinations: a predictable yet avoidable symptom of the fundamental causes of inequality. Humanit Soc Sci Commun.

[21] Suárez-Álvarez A, López-Menéndez AJ (2022). Is COVID-19 vaccine inequality undermining the recovery from the COVID-19 pandemic?. J Glob Health.

[22] Obarisiagbon OE, Mokogwu N (2022). Assessment of knowledge, attitude and factors influencing uptake of COVID-19 vaccine among traders at Edaiken Market, Uselu, Benin City, Edo State, Nigeria. West Afr J Med.

[23] World Health Organisation Rolling updates on coronavirus disease (COVID-19).

[24] Mohamed NA, Solehan HM, Mohd Rani MD, Ithnin M, Che Isahak CI (2021). Knowledge, acceptance and perception on COVID-19 vaccine among Malaysians: a web-based survey. PLoS One.

[25] El-Elimat T, AbuAlSamen MM, Almomani BA, Al-Sawalha NA, Alali FQ (2021). Acceptance and attitudes toward COVID-19 vaccines: a cross-sectional study from Jordan. PLoS One.

[26] Shekhar R, Sheikh AB, Upadhyay S, Singh M, Kottewar S, Mir H (2021). COVID-19 Vaccine Acceptance among Health Care Workers in the United States. Vaccines (Basel).

[27] Adane M, Ademas A, Kloos H (2022). Knowledge, attitudes, and perceptions of COVID-19 vaccine and refusal to receive COVID-19 vaccine among healthcare workers in northeastern Ethiopia. BMC Public Health.

[28] Macchia A, Ferrante D, Angeleri P, Biscayart C, Mariani J, Esteban S (2021). Evaluation of a COVID-19 vaccine campaign and SARS-CoV-2 infection and mortality among adults aged 60 years and older in a middle-income country. JAMA Netw Open.

[29] Feldstein LR, Sutton R, Jalloh MF, Parmley L, Lahuerta M, Akinjeji A (2020). Access, demand, and utilization of childhood immunization services: A cross-sectional household survey in Western Area Urban district, Sierra Leone, 2019. J Glob Health.

[30] Kohon J (2018). Social inclusion in the sustainable neighborhood? Idealism of urban social sustainability theory complicated by realities of community planning practice, City, culture and society.

